# Everybody's Page

**Published:** 1889-07-06

**Authors:** 


### Everybody's Page.

There is some question in Germany of imposing a tax on saccharin, as it appears that saccharin and grape sugar are likely to be used for the adulteration of cane sugar.

Surgery is more honoured in Italy than in England, if we may judge from the fact that in the Anatomical Theatre at Bologna a memorial tablet has been set up in commemoration of Prof. Pietro Loreta's first performance of the operation of resection of the liver, on the 26th August, 1887.

Pity the poor adulterator! He had manufactured some beautiful coffee-berries, which were selling largely in a shop at Dortmund, in Holland, when the police, on the recommendation of the official analyst, came and confiscated them. Is there no place where a man can earn a dishonest living!

The editor of the *New England Medical Monthly* has a sharp pen, and a low opinion of his fellows. He recently published the following statement: "We believe that there are no people or profession in the world that make such fools of themselves as do doctors, either alone, individually, or collectively as societies."

Are athletics and scholarship antagonistic? Prof. Richards, of Yale College, Connecticut, has studied the records of 2,425 students in order to determine this point, and has come to the conclusion that the athletes do not stand so high in scholarship as the non-athletes, but that they do not fall so far behind as to afford sufficient reason for discouraging physical exercise. There are some students, indeed, who excel both in brain and muscle, but even if we do not want these, the intelligence of the average athlete seems to be sufficiently good; and perhaps if Prof. Richards could follow his students' careers through life, he would find even greater reason for approving of a moderate amount of athletic development.

Dr. G. F. Brush, a New York physician, has recently formulated a theory that connects tuberculous disease in man with that among cattle. It has long been known that the bovine race is pre-eminently tuberculous, but the general impression has been that owing to the great difference in normal temperature, the disease was not transmitted to the human race. Dr. Brush, however, has collected a large number of statistics that seem to prove that wherever cattle are common, and dairy produce forms a regular part of the food of the people, phthisis is common, while the absence of the dairy cow is coincident with the absence of phthisis. Dr. Brush's statistics are interesting, though it cannot be regarded as certain that he is right in his theory of the causation of phthisis.

What shall we do with our old clothes? Many of us dislike to have dealings with the professional purchaser of wardrobes, and if we present our "cast-offs" to servants we may have the pleasure of hearing Jane confide to Sarah her contempt for the gift. "Does missus expect *me* to wear that old-fashioned thing that she's had herself for two years!" or something of the sort. It is an unfortunate characteristic of human nature that people despise a thing that is given, when they would value the same thing if it were purchased. A good work has been done in many parishes of late years by the sale by auction of cast-off clothes. These are bought by the best class of the poor, those who know the value of a good material and are not particular about fashion. The money thus made may be expended in various ways. In one parish it was spent on a cripples' holiday; in another, a crèche, which relieved over 5,000 children in the course of last winter, was mainly kept up by it.

Dr. Franz Heller, o Vienna, has discovered the infallible specific against sea-sickness. It consists in keeping the body level with the motion of the ship, bending the right knee as the vessel rises to that side, and the left knee when the motion is reversed. Very good, Dr. Heller, but how long could mortal man keep up the treatment? One's limbs would ache a little between, say, London and Melbourne, if one were constantly bending one knee or another.

A letter in a medical contemporary to the effect that papaïn has afforded marked relief in a case of cancer has caused a sudden demand for the drug, though the writer does not give his name, and leaves to the medical profession at large the onus of confirming his experience. As a matter of fact, Messrs. Christy and Co., who introduced the drug to this country, have been recommending it for some time in cases of a cancerous nature, and Dr. E. Hurry Fenwick has had most satisfactory results from its use.

Dr. Lander Brunton, F.R.S., has just published his Croonian lectures on the "Relationship between Chemical Structure and Physiological Action." Ordinary people have very hazy ideas about chemical structure, and sometimes on that account fail to appreciate important practical points. Decompositions that are commonly taking place in meat, in milk, in beer, and in various other food stuffs, often appear of very small consequence to the uninstructed, whereas they may be fraught with danger, even to life itself. Dr. Brunton expounds and illustrates one important series of chemical changes so simply that a child could understand at least the main drift of his teaching. Addressing an audience of physiologists, he says: "To make the subject of chemical constitution clearer, I may perhaps be allowed to make use of a very homely simile. I have here a pocket-knife with a number of blades. At present these blades are all shut, and the knife may b given to a child to play with, or carried in the pocket without the least danger. If I open one of the blades, however, the case is very different. The composition of the knife is exactly the same as before, but by altering the relation of the parts to one another I have converted a harmless plaything into a dangerous weapon. In the case that I have just referred to, of the compound of nitrogen and carbon" (nitrogen and carbon can unite with each other in different proportions) "an alteration in the relation of its parts has a similar effect to the opening of the knife, and converts the comparatively harmless *nitrile* into the deadly *iso-nitrile* or *carbylamine*. Again, while I keep the same handle to my knife, I may alter its use by opening out a boot-hook, a gimlet, or a file; and in a similar way we may alter the functions of an organic radical, which we may compare to the haft of the knife, by attaching to it an atom of amidogen, chlorine, or hydroxyl. If I wish to cut through a piece of cord or something else out of my reach with my pocket-knife, I may effect my purpose by tying my knife to a piece of stick, and, if that be not long enough, by attaching another, and yet another piece of stick, until I have got my handle sufficiently long for my purpose. Such a process as this may be compared to adding on carbon atom to carbon atom in a lengthening chain as in the higher paraffins; but just as my shank would by-and-by become too heavy and cumbrous to allow me to handle it, and thus become useless for my purpose, so we find that the higher paraffins, although from their high molecular weight they ought to be powerful anæsthetics, are too sparingly absorbed to have any physiological action at all, and, instead of producing either intoxication or anæsthesia, like marsh gas, are simply used under the name of "Vaseline" to protect the skin.

